# Myeloperoxidase inhibition may protect against endothelial glycocalyx shedding induced by COVID-19 plasma

**DOI:** 10.1038/s43856-023-00293-x

**Published:** 2023-05-05

**Authors:** Andrew Teo, Louisa L. Y. Chan, Christine Cheung, Po Ying Chia, Sean Wei Xiang Ong, Siew Wai Fong, Lisa F. P. Ng, Laurent Renia, David Chien Lye, Barnaby Edward Young, Tsin Wen Yeo

**Affiliations:** 1 Lee Kong Chian School of Medicine, Nanyang Technological University, Singapore, Singapore; 2 Department of Medicine, The Doherty Institute, University of Melbourne, Melbourne, VIC Australia; 3 National Centre for Infectious Diseases, Singapore, Singapore; 4 Institute of Molecular and Cell Biology, Agency for Science and Technology and Research (A*STAR), Singapore, Singapore; 5 Department of Infectious Diseases, Tan Tock Seng Hospital, Singapore, Singapore; 6 A*STAR Infectious Diseases Lab (A*STAR ID Labs), Agency for Science, Technology and Research (A*STAR), 8A Biomedical Grove, Immunos #05-13, Singapore, 138648 Singapore; 7 Institute of Infection, Veterinary and Ecological Sciences, University of Liverpool, Liverpool, UK; 8 National Institute of Health Research, Health Protection Research Unit in Emerging and Zoonotic Infections, University of Liverpool, Liverpool, UK; 9 School of Biological Sciences, Nanyang Technological University, Singapore, Singapore; 10 Yong Loo Ling School of Medicine, National University of Singapore, Singapore, Singapore

**Keywords:** Viral infection, Neutrophils, Respiratory distress syndrome

## Abstract

**Background:**

SARS-CoV-2, the causative agent of COVID-19, is a threat to public health. Evidence suggests increased neutrophil activation and endothelial glycocalyx (EG) damage are independently associated with severe COVID-19. Here, we hypothesised that an increased level of blood neutrophil myeloperoxidase (MPO) is associated with soluble EG breakdown, and inhibiting MPO activity may reduce EG damage.

**Methods:**

Analysing a subset of acute and convalescent COVID-19 plasma, 10 from severe and 15 from non-severe COVID-19 cases, and 9 from pre-COVID-19 controls, we determined MPO levels, MPO activity and soluble EG proteins (syndecan-1 and glypican-1) levels by enzyme-linked immunosorbent assay. In vitro primary human aortic endothelial cells were cultured with plasma untreated or treated with specific MPO inhibitors (MPO-IN-28, AZD5904) to determine EG shedding. We then investigated whether inhibiting MPO activity decreased EG degradation.

**Results:**

In COVID-19 plasma, MPO levels, MPO activity and levels of soluble EG proteins are significantly raised compared to controls, and concentrations increase in proportion to disease severity. Despite clinical recovery, protein concentrations remain significantly elevated. Interestingly, there is a trend of increasing MPO activity in convalescent plasma in both severe and non-severe groups. MPO levels and MPO activity correlate significantly with soluble EG levels and inhibiting MPO activity leads to reduced syndecan-1 shedding, in vitro.

**Conclusions:**

Neutrophil MPO may increase EG shedding in COVID-19, and inhibiting MPO activity may protect against EG degradation. Further research is needed to evaluate the utility of MPO inhibitors as potential therapeutics against severe COVID-19.

## Plain language summary

COVID-19 can result in severe disease and is potentially fatal. Neutrophils, the most abundant white blood cells in circulation, secrete antimicrobials that have been linked to severe COVID-19 development. The endothelial glycocalyx (EG) is a carbohydrate rich layer that coats the inner surface of the vasculature and damage to the EG is observed in severe COVID-19. Here, we investigate whether myeloperoxidase, an antimicrobial released by neutrophils, is associated with EG damage in COVID-19 patients. We also determine whether reducing myeloperoxidase activity prevents damage to the EG. Our results suggest myeloperoxidase is associated with EG damage and severe COVID-19. We also demonstrated that a reduction in myeloperoxidase activity may protect against EG degradation. Further studies to evaluate the utility of MPO inhibitors as a therapy against severe COVID-19 are warranted.

## Abstract (web summary)

Teo et al. study neutrophil activity and endothelial glycocalyx damage in plasma collected from healthy individuals and patients with severe and non-severe COVID-19. They show that neutrophil myeloperoxidase activity and endothelial glycocalyx proteins increase with disease severity, and remain high despite clinical recovery.

## Introduction

Coronavirus disease 2019 (COVID-19), caused by the highly contagious severe acute respiratory syndrome coronavirus 2 (SARS-CoV-2), is a potentially life-threatening disease to some individuals^1^. Clinical observations associated with severe outcomes include acute respiratory distress syndrome, a leading cause of COVID-19 mortality^2^. Despite global efforts in delineating mechanisms of severe COVID-19, the precise pathological pathway remains elusive.

Strong evidence suggests aggravated immune responses contribute to severe COVID-19^3,4^. Detailed examination, in adults, revealed higher neutrophil to lymphocyte ratio, and increased neutrophil numbers and neutrophil degranulation to be associated with severe disease^3,5,6^. Consistent with autopsy observations of neutrophils infiltration and presence of neutrophil extracellular trap formation in lung specimens of patients who succumbed to COVID-19^7,8^. SARS-CoV-2 can activate neutrophils to release myeloperoxidase (MPO), a leukocyte heme-enzyme, and increased peripheral MPO levels were observed in COVID-19 ICU patients^3,9^. However, whether functional active MPO correlates with severe COVID-19 has not been reported. MPO promotes neutrophil recruitment, cytokine production, and MPO catalysed H_2_O_2_ to generate hypochlorous acid, which is capable of microbial killing and host tissue damage^10^. Although the link between MPO and severe disease is complex, it is likely that increased MPO levels and activity promote inflammation contributing to COVID-19 pathology.

The endothelial glycocalyx (EG) coats the luminal surface of the endothelium promoting vascular integrity and homeostasis^11^. Increased EG degradation and endothelial damage have been associated with severe COVID-19 associated mortality, and it is proposed that EG degradation precedes endothelial injury^12,13^. Pro-inflammatory mediators including cytokines and extracellular matrix sheddases promote EG breakdown, and inhibitors that target these proteins, in vitro, were associated with reduced EG degradation^14–16^. MPO was previously demonstrated to partake in EG damage^17^. Furthermore, in other pandemic viruses such as influenza virus, MPO inhibition was associated with reduced endothelial damage in lung specimen of influenza-infected mice^18,19^. However, whether MPO is involved in EG degradation in COVID-19 has not been investigated.

To address these research gaps, we hypothesize that increased MPO levels and MPO activity are associated with increased EG breakdown, and inhibiting MPO activity may reduce EG degradation. Here, we demonstrate increased MPO levels, MPO activity and EG breakdown to be associated with severe pathology, and protein levels remain elevated despite clinical recovery. Importantly, MPO levels and MPO activity correlate with EG breakdown, and reducing MPO activity may protect against EG degradation.

## Methods

### Study participants

A subset of randomly selected COVID-19 positive samples from an earlier study was used in the present study^5^. Acute and convalescent plasma were collected. The samples were categorised into non-severe (*n* = 15) and severe COVID-19 (*n* = 10) groups, previously defined^5^. Additionally, a pre-COVID-19 healthy control group (*n* = 9) was included.

### Cell cultures

Primary human aortic endothelial cells (HAEC, Lonza), 1 × 10^5^ cells/ml, were cultured on Rat-tail Collagen I (Thermo Fisher Scientific) coated T-75 flasks (Corning) and maintained in EGM2-MV (Lonza) supplemented with 10% foetal bovine serum (Thermo Fisher Scientific) in 5% humidified CO_2_ incubator at 37 °C until confluent. For experimentation, HAEC was trypsinised from T-75 flask, added into six wells culture plate at 1 × 10^5^ cells/ml and cultured overnight. Cells confluency was ensured before experimentations.

### Enzyme-linked immunosorbent assay

Plasma and supernatants (from HAEC experimentations, described below) levels of MPO, syndecan-1 and glypican-1 (both soluble EG proteins), were quantified at 1:10 dilution using Human DuoSet ELISA based on manufacture’s protocols. Standard curves were generated to determine protein concentrations. Absorbance at 450 nm was determined using BioTek EPOCH 2 plate reader.

### Myeloperoxidase activity assay

MPO enzyme activity was determined with MPO colorimetric activity assay kit (ab105136, Abcam Lot#GR3445894-2) according to manufacturer’s protocol. Plasma samples (1:10 dilution) were either untreated or treated with 10 μM MPO inhibitors (details next section), followed by 60 mins incubation at room temperature. Absorbance at 412 nm was determined using BioTek EPOCH 2. MPO activity is represented as the amount of MPO required to generate taurine chloramine to consume 1.0 μmol of DTNP per minute at room temperature.

### HAEC experimentations

HAEC was subjected to various conditions to determine syndecan-1 and glypican-1 shedding and whether MPO inhibition abrogate soluble EG shedding. MPO activity were inhibited with 10 μM MPO inhibitor 28 (MPO-IN-28; Lot#41855, MedChemExpress)^20^ or 10 μM AZD5904 (MedChemExpress; Lot#28433)^8,21^. Note, inhibitor was diluted in DMSO.

HAEC experimentations were performed with slight modifications^14,15^. Confluent HAEC (1 × 10^5^ cells) in six-well plates (Corning) were serum starved for six hours, followed by no treatment (control) or treatment with: 1) five severe COVID-19, five non-severe COVID-19 and three pre-COVID-19 plasma, tested individually at 10% concentration (samples random selection); 2) MPO inhibitors co-incubated with plasma [same as condition 1]; 3) 50 nM purified recombinant MPO (ab91116, Abcam) with 30 μM H_2_O_2_; and 4) MPO inhibitors [MPO-IN-28 or AZD-5904] co-cultured with 50 nM purified MPO and 30 μM H_2_O_2_. The cells were then incubated in 5% humidified CO_2_ incubator at 37 °C for 16 h, and supernatant was subsequently collected to assay for syndecan-1 and glypican-1 levels by ELISA. Data generated for all conditions were based on two independent experimentations, and a third experimentation was conducted for conditions 3 and 4. Of note, only convalescent samples were used because acute samples were subjected to virus inactivation procedures based on required regulations from Singapore Ministry of Health^5^. Cells passages between 8-10 were used.

### Statistics and reproducibility

Plasma to quantify proteins levels were tested in duplicates. Two independent HAEC experiments were performed to obtain data for conditions 1-4 described above. A third experiment was conducted for conditions 3-4 to generate triplicate values.

Categorical variables were assessed using χ^2^ tests. Kruskal–Wallis (nonparametric) and Student *t*- tests (parametric) were performed on continuous variables. Paired continuous variables and plasma protein concentrations were evaluated using paired *t*-test and Wilcoxon singed rank test. Correlations were performed with Spearman correlation. *P* < 0.05 was considered statistically significant.

Data obtained were analysed using STATAv16 (StataCorp). Graphical representations were done in Prism (GraphPad v9).

### Reporting Summary

Further information on research design is available in the Nature Portfolio Reporting Summary linked to this article.

## Results

### Participants’ characteristics

A subset of plasma collected at National Centre for Infectious Diseases, Singapore, from a larger study was used here^5^. The samples were categorised into non-severe (*n* = 15) and severe COVID-19 (required oxygen supplementation, *n* = 5, and ICU-admitted, *n* = 5). The baseline characteristics of subjects at enrolment are presented in Table 1. Briefly, acute samples were collected on median day 6 (non-severe) and median day 11 (severe) post-onset of symptoms. Convalescent samples were collected between day 33-34 post-onset of symptoms. In the severe COVID-19 group, subjects were significantly older (median: 55.5 years) and a higher proportion (50.0%) had hypertension compared to non-severe group (44.0 years; 13.3%). Additionally, neutrophil counts were significantly higher in the severe group (4.77 × 10^3^/μL) compared to non-severe group (2.40 × 10^3^/μL).

**Table 1:** Study population baseline characteristics at enrolment, length of hospitalisation and timeline of samples collection.

Variables	Non-severe (*n* = 15)	Severe (O2 and ICU, *n* = 10)	*P* value	Pre-Covid controls, (*n* = 9)
*Demographics*				
Age, median (IQR)	44 (31–54)	55.5 (47–63)	0.049	65 (45–66)
Gender, male (%)	13 (86.7)	8 (80%)		
Ethnicity, Chinese (%)	7 (46.7)	5 (50.0)		
*Comorbidities*				
Hypertension (%)	2 (13.3)	5 (50.0)	0.045	
Hyperlipidaemia (%)	1 (6.7)	6 (60.0)	0.004	
*Clinical parameters*				
C-reactive protein, median (IQR)	2.8 (1.6–9.3)	70.6 (32.5–114.6)	<0.0001	
Haemoglobin, mean (SD)	15.1 (0.87)	14.4 (1.48)		
Haematocrit, mean (SD)	44.5 (2.2)	42.4 (3.8)		
Lymphocytes, median (IQR)×10^3^/μL	1.35 (0.93 – 1.71)	0.94 (0.79 – 1.23)		
Monocytes, median (IQR)×10^3^/μL	0.58 (0.48–0.64)	0.50 (0.03–0.53)		
Neutrophils, median (IQR)×10^3^/μL	2.40 (2.06–3.59)	4.77 (2.72–5.02)	0.005	
Platelets, median (IQR)×10^3^/μL	199.0 (159.0– 250.0)	225.5 (178.0–285.0)		
White blood cells, median (IQR) 10^3^/μL	4.80 (3.50–5.40)	6.15 (4.50–6.40)		
Neutrophils-to-Lymphocytes	1.57 (1.17–2.76)	4.39 (2.42–5.96)	0.01	
*Hospital stay length (days)*				
Hospitalisation length, median (IQR)	10.0 (9.0–17.0)	15.5 (13.0–19.0)	0.02	
Days in ICU, median (IQR)		1 (0.0–6.0)		
*Samples collection days post-symptoms/hospital stay (days)*				
Acute samples, days after first detection of symptoms, median (IQR)	6.0 (4.0–7.0)	11.0 (7.0–12.0)	0.003	
Recovery samples, days after first detection of symptoms, median (IQR)	34.0 (30.0–35.0)	33.5 (30.0–35.0)		
Recovery samples, days after hospital discharged, median (IQR)	20.0 (18.0–22.0)	10.5 (8.0–16.0)	0.01	

Data are median (interquartile range) or no. (%), unless otherwise stated. *P* values of <0.05 were consider statistically significant. *IQR* Interquartile range, *ICU* Intensive care unit.

### Plasma myeloperoxidase levels are increased in COVID-19

**Fig. 1:**
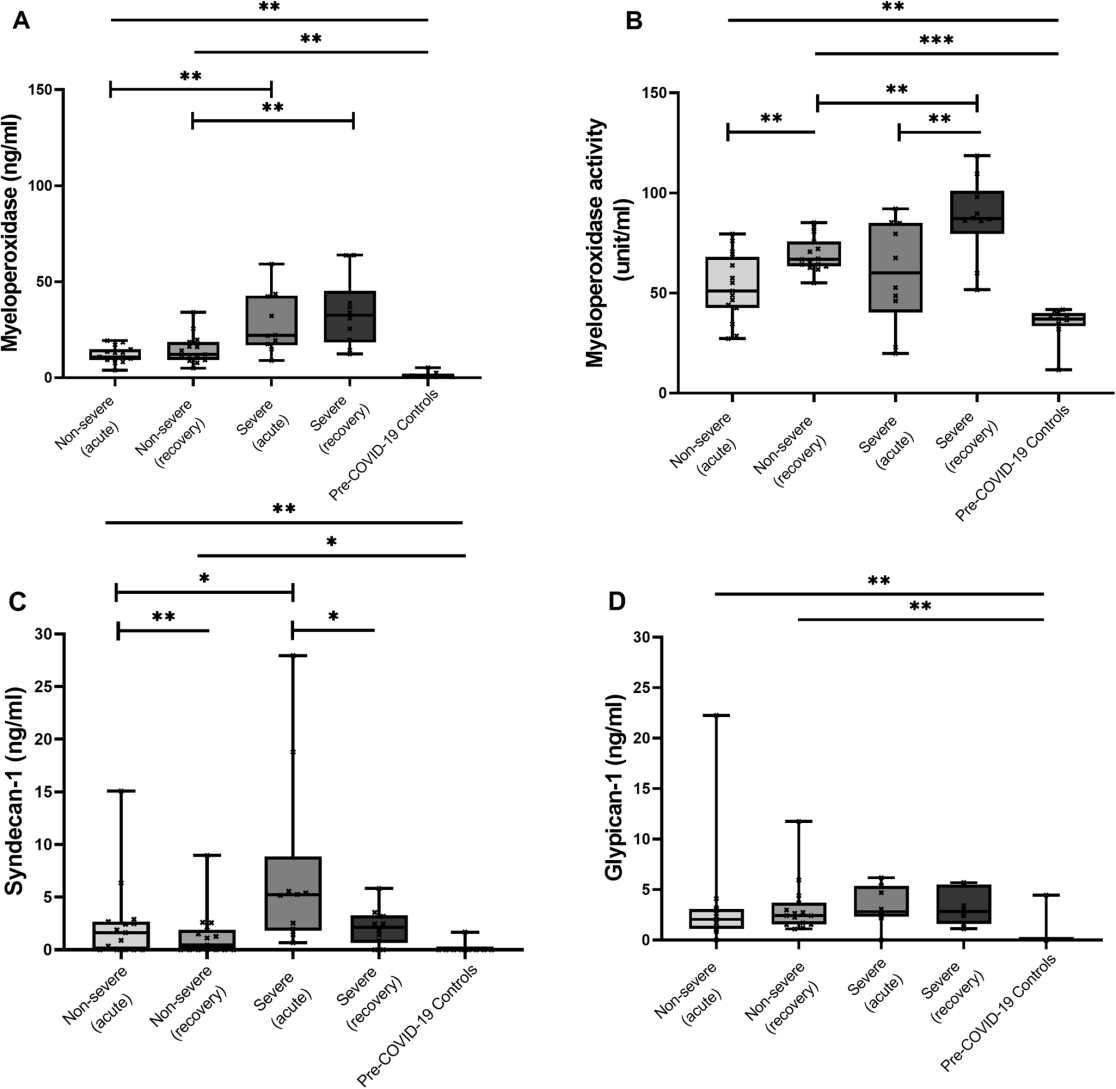
Blood concentrations of myeloperoxidase (MPO), MPO activity, syndecan-1 and glypican-1 in COVID-19 subjects at acute and convalescent phases, and controls. Non-severe (*n* = 15), severe (*n* = 10) and pre-COVID-19 controls (*n* = 9). **A** MPO concentrations; **B** MPO activity; **C** Syndecan-1 concentrations; **D** Glypican-1 concentrations. Data presented in box and whiskers plots demonstrating median and interquartile range, and whiskers representing 10-90 percentiles. **A, C, D** Protein concentrations were extrapolated from serially diluted standard curves presented in ng/ml. MPO activity: amount of MPO required to generate taurine choloramine to consume 1.0 μmol of DTNB probe per minute at room temperature (incubated for 60 min) presented in unit/ml. Solid lines across groups; bars between groups. ^*^P < 0.05, ^**^P < 0.01, ^***^P < 0.001 by the Kruskal–Wallis test and Wilcoxon sign rank test.

At acute and convalescent phases, COVID-19 subjects had significantly higher plasma MPO levels compared with controls [controls, median (IQR); 5.74 (5.51–11.61) ng/ml], both time points (*p* < 0.01). Additionally, plasma MPO levels were also significantly increased in severe [acute: 22.04 (17.54–42.37) ng/ml; convalescent: 32.50 (19.70–38.98) ng/ml] compared with non-severe cases [acute: 10.93 (9.32–14.80) ng/ml; convalescent: 12.17 (9.25–18.58) ng/ml], at both time points (*p* < 0.01). Despite clinical recovery, a trend of increasing MPO concentrations between acute and convalescent was observed in severe cases; however, no statistical significance was reached (Fig. 1A). Lastly, we observed MPO levels to be significantly correlated to neutrophil count (R = 0.54, *p* < 0.01) (Supplementary Table 1).

### Plasma MPO activity are elevated in COVID-19

**Fig. 2:**
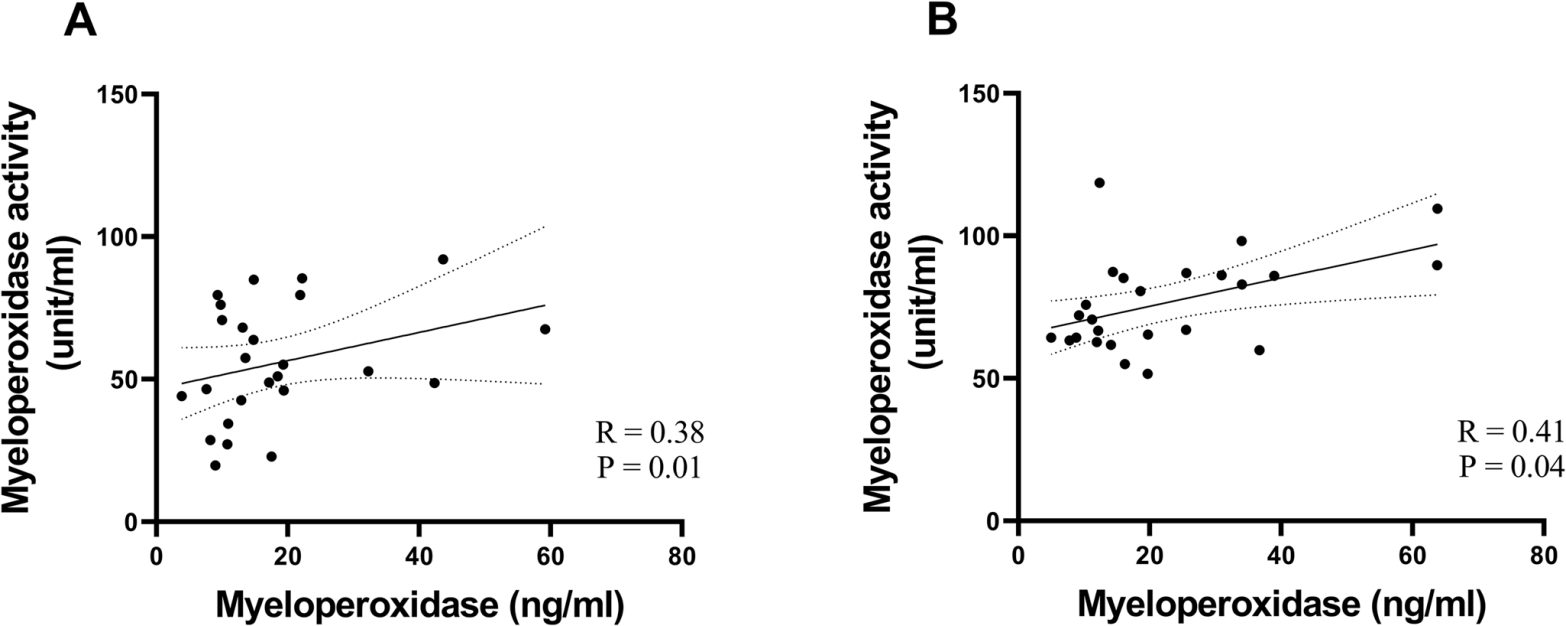
Correlations between myeloperoxidase levels and myeloperoxidase activity at acute and convalescent phases. Non-severe (*n* = 15) and severe (*n* = 10) **A** Acute phase; **B** Convalescent phase. Rho and *P* value determined by spearman correlation tests.

We then evaluated MPO activity. At acute and convalescent phases, plasma MPO activity were significantly higher in COVID-19 subjects compared with controls [controls, median (IQR); 36.99 (34.80–39.01) unit/ml, acute (*p* < 0.01); convalescent (*p* < 0.001)]. Similarly, despite clinical recovery, a trend of increasing MPO activity in both non-severe [acute: 51.01 (42.68–68.12) unit/ml; convalescent: 66.77 (63.33–75.82) unit/ml, *p* < 0.01] and severe [acute: 60.16 (46.03–84.89) unit/ml; convalescent: 60.16 (46.03–84.89) unit/ml, *p* < 0.01] groups were observed, (Fig. 1B). Additionally, quantitative MPO levels correlated positively with MPO activity, acute (R = 0.38, *p* < 0.05) convalescent (R = 0.41, *p* < 0.05) (Fig. 2A, B).

### Inhibiting myeloperoxidase activity with inhibitors

**Fig. 3:**
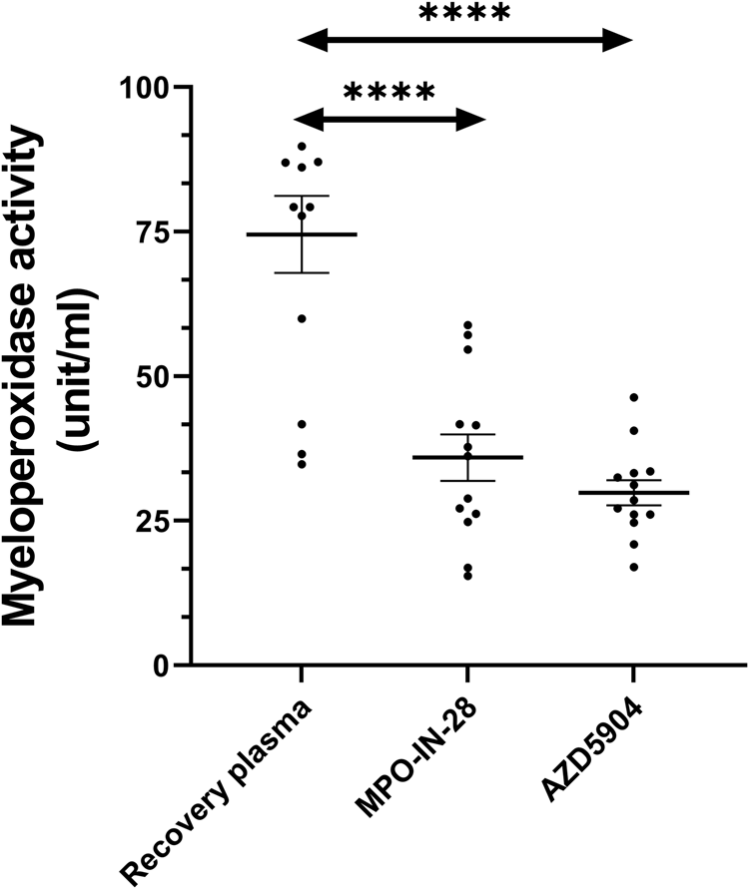
Plasma myeloperoxidase (MPO) activity and treatment with MPO inhibitors. Data generated with COVID-19 recovery samples comprising of severe COVID-19 (*n* = 5), non-severe (*n* = 5); and controls (*n* = 3). Plasma MPO activity was determined in untreated or treated with MPO-inhibitors (MPO-inhibitor-28, 10 μM and AZD-5904, 10 μM). ^****^P < 0.001 by paired-*t* test. Data presented in scatter plot, horizontal lines represent mean and ±(S.E.M).

Because of higher MPO activity observed in convalescent samples, we then sought to explore on the effectiveness of MPO-IN-28 and AZD-5904 in supressing MPO activity. We randomly selected 13 samples consisting of five non-severe, five severe and three controls, and treated samples with 10 μM of inhibitors^22^. Samples treated with MPO-IN-28 [mean (SD): 35.92 (14.48) unit/ml] or AZD-5904 [29.82 (7.80) unit/ml) demonstrated an approximate 51–59% decrease in MPO activity compared to non-treated samples [68.75 (22.54) unit/ml], both *p* < 0.0001 (Fig. 3). These samples were then used to explore on the association of MPO activity inhibition on EG degradation, described later.

### Increased soluble endothelial glycocalyx breakdown in COVID-19

At acute and convalescent phases, COVID-19 positive subjects had significantly higher syndecan-1 [acute (*p* < 0.01); convalescent (*p* < 0.05)] and glypican-1 [acute and convalescent both *p* < 0.01)] breakdown compared with controls [both proteins below limit of detection]. At acute phase, comparing between severe and non-severe groups, syndecan-1 levels [median (IQR), severe: 5.22 (1.97–5.57) ng/ml; non-severe: 1.64 (0.00–2.66) ng/ml, *p* < 0.05] but not glypican-1 levels [severe:2.81 (2.36–5.34) ng/ml; non-severe: 2.05 (1.10–3.06) ng/ml, *p* = 0.13] were significantly raised in severe COVID-19 group. At convalescent, no significant differences in soluble EG proteins were observed between both groups. Additionally, syndecan-1 levels at convalescent were significantly lower compared to enrolment samples in the severe and non-severe groups, *p* < 0.05 and *p* < 0.01, respectively (Fig. 1C, D).

### Correlates of inflammatory mediators and glycocalyx shedding in COVID-19

**Fig. 4:**
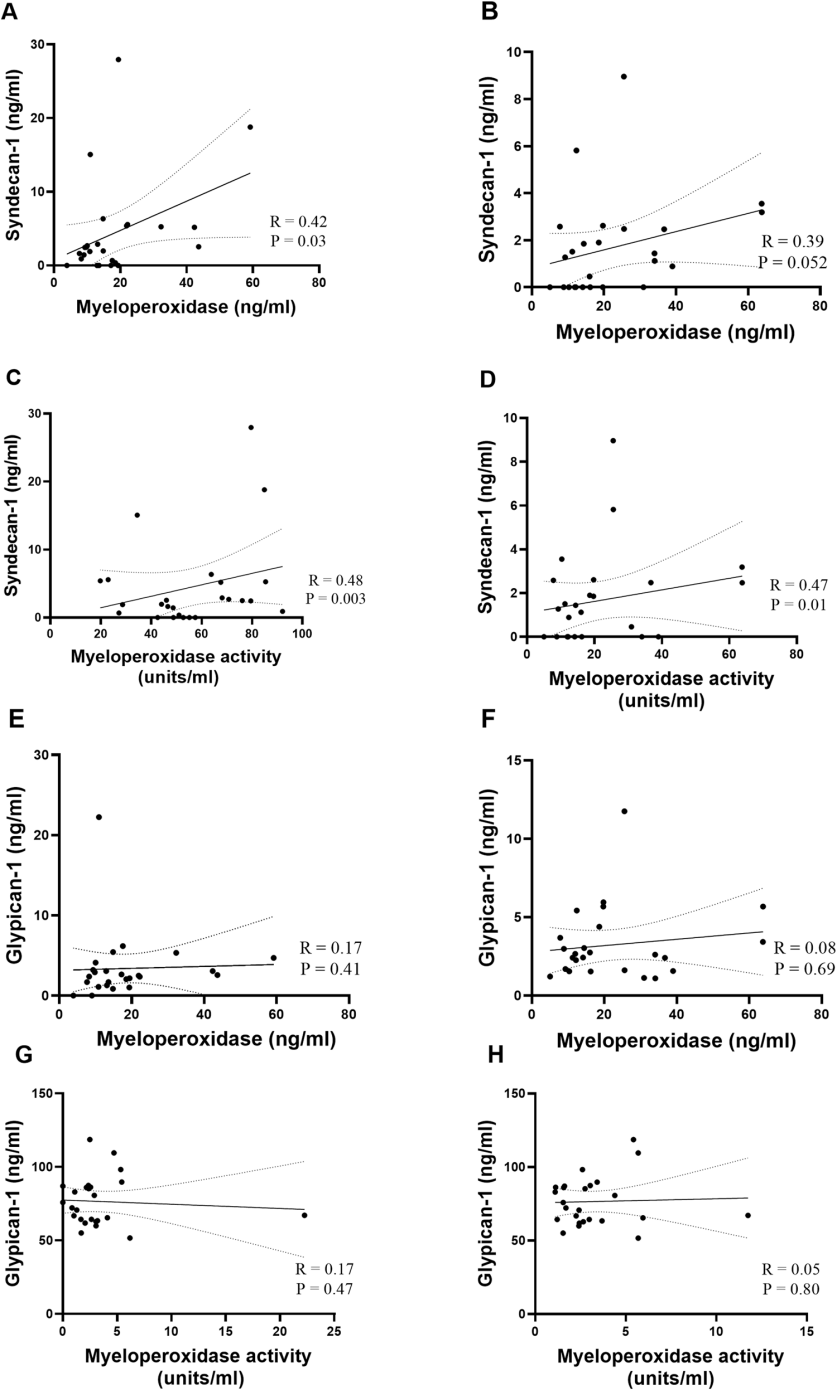
Correlations between myeloperoxidase (MPO) levels, MPO activity and soluble glycocalyx proteins at acute and convalescent phases. Non-severe (*n* = 15), severe (*n* = 10) and pre-COVID-19. **A** Acute (syndecan-1 vs MPO levels); **B** Convalescent (syndecan-1 vs MPO levels); **C** Acute (syndecan-1 vs MPO activity); **D** Convalescent (syndecan-1 vs MPO activity); **E** Acute (glypican-1 vs MPO levels); **F** Convalescent (glypican-1 vs MPO levels); **G** Acute (glypican-1 vs MPO activity); **H** Convalescent (glypican-1 vs MPO activity). Rho and *P* value determined by spearman correlation tests.

In this cohort of COVID-19 plasma (*n* = 25), we observed positive correlations between MPO levels and syndencan-1 shedding at both acute (R = 0.42, *p* = 0.03) and convalescent (R = 0.39, *p* = 0.052) phases (Fig. 4A, B). Additionally, MPO activity was positively associated with syndecan-1 shedding, acute (R = 0.48, *p* = 0.003) and convalescent (R = 0.47, *p* = 0.01) (Fig. 4C, D). In contrast MPO levels and MPO activity were less associative with glypican-1 shedding, [acute: R = 0.17, *p* = 0.47; convalescent R = 0.05, *p* = 0.80] (Fig. 4E–H).

We then extended the investigation to other previously measured inflammatory mediators on their impact on EG shedding^5^. At the acute phase, CRP and IP-10 levels correlated positively to syndencan-1 levels, whereas only IP-10 correlated with glypican-1 shedding. In contrast, IL-10 levels had a negative relationship with syndecan-1 levels. At the convalescent phase, IL-6 levels were positively associated with syndecan-1 shedding and IL-2 levels were negatively associated with glypican-1 shedding (Supplementary Table 2).

### Myeloperoxidase inhibition on EG degradation

**Fig. 5:**
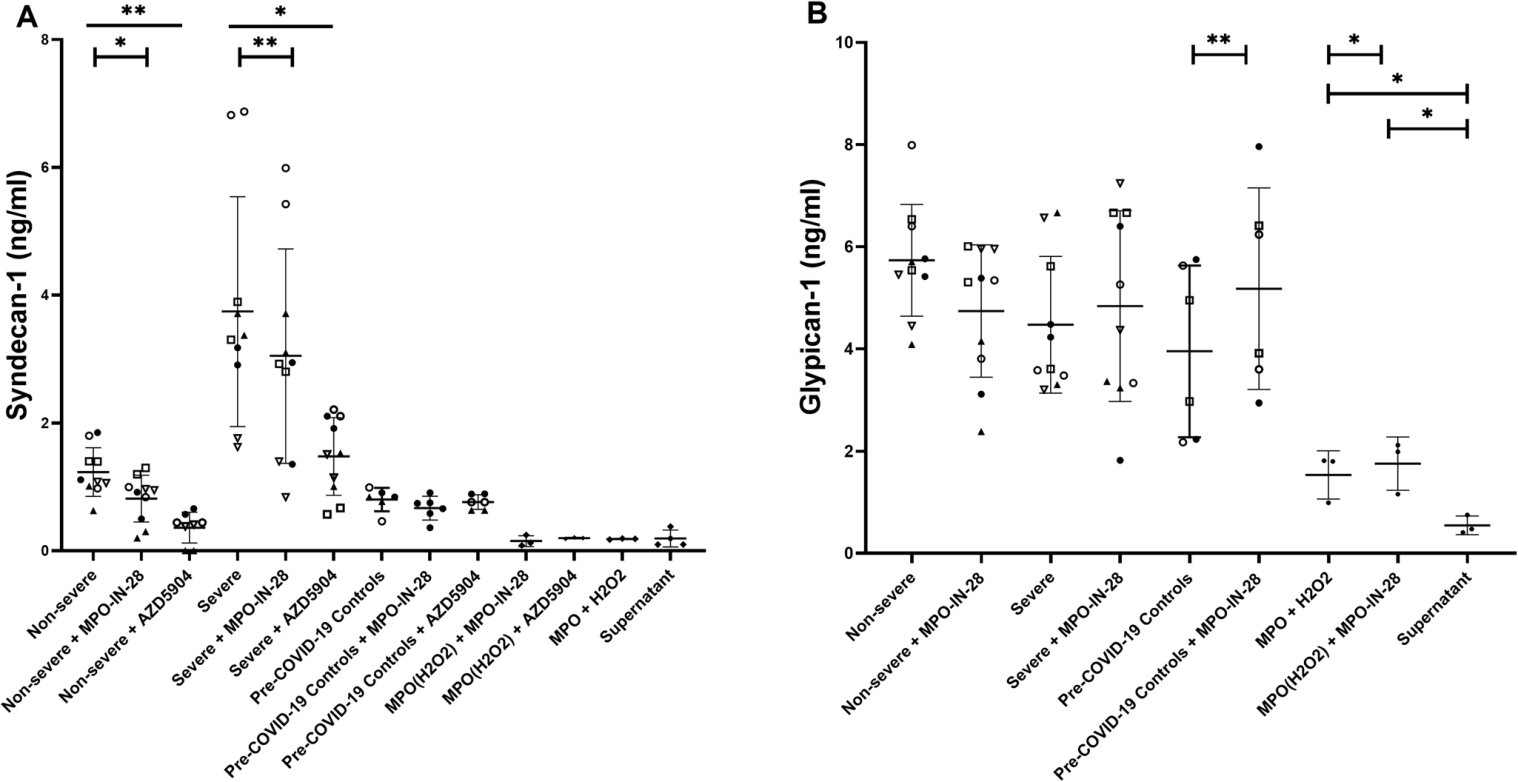
Convalescent COVID-19 plasma induced endothelial glycocalyx shedding and myeloperoxidase (MPO) inhibition reduced shedding. **A**, **B** Graphical representative of cleaved syndecan-1 and glypican-1 (ng/ml) from human aortic endothelial cells treated with convalescent COVID-19 plasma [uncomplicated (*n* = 5) and complicated (*n* = 5)], control plasma (*n* = 3)], purified MPO (50 μM) with hydrogen peroxide (30 μM), in the presence or absence of an irreversible MPO-inhibitors (MPO-inhibitor-28, 10 μM and AZD-5904, 10 μM), after 16 h. Data are representative of two independent experiments. Middle solid lines are mean ± SD. Protein concentrations were extrapolated from serially diluted standard curves. Solid lines: measurements across groups; bars: measurements between groups. ^*^P < 0.05, ^**^P < 0.01 by paired-*t* test. Individual samples were randomly picked. Note, only 4 samples from uncomplicated group were available for MPO inhibition with AZD5904. Untreated convalescent plasma syndecan-1 levels [mean (SD); non-severe 0.35 (0.55) ng/ml; severe 2.34 (1.39) ng/ml].

In vitro, HAEC treated with convalescent plasma demonstrated increased syndecan-1 shedding [mean (SD), non-severe: 1.23 (0.28) ng/ml; severe: 3.66 (1.73) ng/ml; and controls: 0.80 (0.07) ng/ml, *p* < 0.01]. When convalescent plasma was incubated with HAEC treated with MPO-IN-28, we observed reduced sydencan-1 shedding (non-severe: 0.73 (0.51) ng/ml, *p* = 0.05; severe: 3.13 (1.82) ng/ml, *p* = 0.006; controls: 0.48 (0.17) ng/ml, *p* = 0.06) compared with untreated plasma. Similar but more profound reduction in syndecan-1 shedding was observed in convalescent plasma incubated with AZD5904 (non-severe: 0.36 (0.26) ng/ml, *p* = 0.001; severe: 1.48 (0.64) ng/ml, *p* = 0.02; controls: 0.73 (0.11) ng/ml, *p* = 0.06) compared with untreated plasma. Note, only four samples from non-severe group were available for AZD5904 treatment [mean, *n* = 4, syndecan-1: 1.23 (0.31) ng/ml]. Interestingly, MPO-H_2_O_2_ catalysation or inhibiting MPO-H_2_O_2_ catalysation did not impact on syndecan-1 shedding [MPO-H_2_O_2_ + IN-28; 0.15 (0.09) ng/ml or AZ5904; 0.18 (0.01) ng/ml; MPO-H_2_O_2_; 0.19 (0.004) ng/ml] compared with HAEC supernatant [0.19 (0.13) ng/ml] (Fig. 5A).

On the other hand, when HAECs were incubated with untreated plasma, higher glypican-1 shedding was observed in the non-severe group [5.93 (0.92) ng/ml] but not in the severe group [4.47 (0.74) ng/ml] compared to the control group [3.95 (0.05) ng/ml], *p* = 0.01 and *p* = 0.28, respectively. Contrast to observations with syndecan-1 shedding, inhibiting MPO activity with MPO-IN-28 did not demonstrate reduce glypican-1 shedding [non-severe, 4.91 (1.31) ng/ml, *p* = 0.14; severe, 4.83 (1.32) ng/ml *p* = 0.69] compared with untreated plasma. Surprisingly, inhibiting MPO with MPO-IN-28 [5.17 (0.26) ng/ml, *p* = 0.01] appeared to enhanced glypican-1 shedding in the control group compared with untreated control plasma. Lastly, MPO-H_2_O_2_ catalysation may induce higher glypican-1 shedding [1.53 (0.47) ng/ml] compared to supernatant control [0.54 (0.18) ng/ml] *p* = 0.01, (Fig. 5B). Of note, we did not compare MPO activity inhibition with AZD5904 on glypican-1 shedding.

## Discussion

In COVID-19 patients, we observed increased plasma MPO levels, MPO activity, syndecan-1 and glypican-1 concentrations to be associated with severe disease. Furthermore, MPO levels and MPO activity correlated positively with soluble EG shedding. We also demonstrated that inhibiting MPO activity with irreversible MPO inhibitors (MPO-IN-28, AZD5904) reduced syndecan-1 shedding, suggesting possible therapeutics that may reduce disease severity.

Increased neutrophil count, especially immature neutrophils, is a strong predictor of severe COVID-19^5^. However, their pathological roles remain unclear. Our observation of increased MPO concentrations in severe COVID-19 cases mirrors findings a Swedish cohort of COVID-19 patients admitted to ICU, and immature neutrophils may be the main source for MPO^3, 23^. Interestingly, despite clinical recovery, MPO levels and MPO activity remained elevated in the severe COVID19 group, and upregulated neutrophils-associated immune signatures including MPO levels was associated with a pulmonary sequela of COVID-19^24^. The cause of this persistent upregulation of neutrophil-associated signatures is unclear; however, residual antigens at recovery may continue to drive inflammation^25^. Together, suggesting that therapeutics to attenuate neutrophil responses should be considered.

Disrupted EG is associated with severe COVID-19^12,14,15^. In agreement, we demonstrated increased syndecan-1 and glypican-1 (albeit lower than syndecan-1 levels) shedding in COVID-19 patients, and protein concentrations were the highest in severe cases. At convalescence, syndecan-1 and glypican-1 shedding is reduced compared with levels measured at enrolment, however, both proteins remain elevated compared with controls, possibly suggesting EG injury that may contribute to endothelial dysfunction despite clinical recovery^26^. To corroborate, in dengue, another viral disease, EG disruption is a strong mediator of endothelial dysfunction^11^. The mechanisms mediating EG shedding is unclear, but earlier evidence and ours suggests pro-inflammatory mediators including CRP, IL-6, IP-10 and MPO to be involved^14,17^. Of relevance, the current standard of care with immunomodulators including corticosteroids, baricitinib and tocilizumab are capable of suppressing inflammation, however, whether treatments reduce EG shedding clinically needs to be investigated^27^. Although in vitro treatment of COVID-19 plasma with low molecular weight heparin (LMWH) and heparan sulfate mimetic minimised glycocalyx perturbation, and LMWH was shown to reduce IL-6 levels in COVID-19 patients^16,28^.

Like previous findings, COVID-19 plasma treatment on primary endothelial cells induced soluble EG shedding, however, differences in syndecan-1 shedding but not glypican-1 disruption was more prominent between COVID-19 severity^14,15^. Differences in endothelial cell types used and differences in cellular attachment between syndecan-1 (transmembrane) and glypican-1 (membrane bound) could explain these observations^11,14^. Of potential importance, inhibiting MPO activity with AZD5904 demonstrated a significant reduction of syndecan-1 shedding, this may protect against EG degradation in the acute and convalescent phases thus reducing the risk of endothelial injury in severe COVID-19^21,26^. AZD5904, is a phase 1 irreversible MPO inhibitor and further in vivo study using suitable airway models to validate its possibility as a therapeutic against severe COVID-19 should be explored^29^. Differences in EG shedding between COVID-19 plasma and purified MPO-H_2_O_2_ catalysation highlight the complexity in MPO induced EG breakdown. However, our data suggests that MPO may act synergistically with other mediators to mediate glycocalyx degradation, and MPO was proposed to bind with heparan sulfate, disrupting the glycocalyx structure, and co-incubation with neutrophils demonstrated syndecan-1 shedding^17^.

There are several limitations in this study. First, plasma instead of bronchoalveolar lavage (BAL) fluid was used to study EG shedding. Whether there are differences in outcomes with BAL samples would need to be evaluated, although blood levels of neutrophil proteins correlated well with disease severity^3,9^. Second, a larger proportion of severe COVID-19 patients had hypertension, which has been associated with EG dysfunction, and hypertension is one of the commonest comorbidities associated with severe COVID-19^30^. Thus, hypertension may confound the increased in soluble EG degradation observed. Nonetheless, elevated sydencan-1 levels have been observed in younger populations with severe COVID-19^31^. Lastly, we could not compare differences in MPO activity in soluble EG shedding between acute and convalescent phases, owing to technical issues. It would be worthwhile to investigate whether there would be differences in MPO-mediated EG shedding at the early onset of disease. The strength of this study is that we used pre-vaccinated samples, reducing potential vaccine-induced confounders, and we determined the functional activity of MPO in COVID-19 subjects compared to previous studies that reported only quantitative levels^3,24^.

## Conclusion

Despite the availability of vaccines, studies that investigate mechanistic causes of severe pathology are urgently needed to design effective therapeutics that can complement vaccinations. This study provide evidence that increased MPO levels to mediate EG shedding and MPO levels remained elevated despite recovery that may contribute to symptoms of long-COVID 19. Inhibiting MPO activity demonstrated possible protection against EG shedding. Future studies on whether dampen of neutrophil responses in COVID-19 improves COVID-19 pathology is urgently needed.

## Supplementary information

Reporting Summary

Description of Additional Supplementary Files

Supplementary Data

Supplementary Information

## Data Availability

The dataset set used for the current study are available from the corresponding author on reasonable request. The source data needed to reproduce Figs. 1 (A–D), 2 (A, B), 3, 4 (A–H) and 5 (A, B) can be found in Supplementary Data.
