# Supplementary material for: Myeloperoxidase inhibition may protect against endothelial glycocalyx shedding induced by COVID-19 plasma
Source: Commun Med (Lond). 2023 May 5;3:62. doi: 10.1038/s43856-023-00293-x (PMC10160718; doi:10.1038/s43856-023-00293-x)
Supplement: Supplementary file 1 — Reporting Summary [file 43856_2023_293_MOESM1_ESM.pdf]

nature portfolio

Corresponding author(s): Andrew Teo

Last updated by author(s): Feb 2, 2023

Reporting Summary

Nature Portfolio wishes to improve the reproducibility of the work that we publish. This form provides structure for consistency and transparency in reporting. For further information on Nature Portfolio policies, see our Editorial Policies and the Editorial Policy Checklist.

Statistics

For all statistical analyses, confirm that the following items are present in the figure legend, table legend, main text, or Methods section.

n/a	Confirmed	
☐	☒	The exact sample size (*n*) for each experimental group/condition, given as a discrete number and unit of measurement
☐	☒	A statement on whether measurements were taken from distinct samples or whether the same sample was measured repeatedly
☐	☒	The statistical test(s) used AND whether they are one- or two-sided<br>*Only common tests should be described solely by name; describe more complex techniques in the Methods section.*
☒	☐	A description of all covariates tested
☒	☐	A description of any assumptions or corrections, such as tests of normality and adjustment for multiple comparisons
☐	☒	A full description of the statistical parameters including central tendency (e.g. means) or other basic estimates (e.g. regression coefficient) AND variation (e.g. standard deviation) or associated estimates of uncertainty (e.g. confidence intervals)
☒	☐	For null hypothesis testing, the test statistic (e.g. *F*, *t*, *r*) with confidence intervals, effect sizes, degrees of freedom and *P* value noted<br>*Give P values as exact values whenever suitable.*
☒	☐	For Bayesian analysis, information on the choice of priors and Markov chain Monte Carlo settings
☒	☐	For hierarchical and complex designs, identification of the appropriate level for tests and full reporting of outcomes
☒	☐	Estimates of effect sizes (e.g. Cohen's *d*, Pearson's *r*), indicating how they were calculated

Our web collection on statistics for biologists contains articles on many of the points above.

Software and code

Policy information about availability of computer code

Data collection	Data tabulated with GraphPad Prism v9, Microsoft Office (Excel)
Data analysis	Data obtained were analysed using STATA v16 (StataCorp). Graphical representations were done in GraphPad Prism v9 (GraphPad software).

For manuscripts utilizing custom algorithms or software that are central to the research but not yet described in published literature, software must be made available to editors and reviewers. We strongly encourage code deposition in a community repository (e.g. GitHub). See the Nature Portfolio guidelines for submitting code & software for further information.

Data

Policy information about availability of data

All manuscripts must include a data availability statement. This statement should provide the following information, where applicable:

- Accession codes, unique identifiers, or web links for publicly available datasets
- A description of any restrictions on data availability
- For clinical datasets or third party data, please ensure that the statement adheres to our policy

The dataset set used for the current study are available from the corresponding authors on reasonable request.

Human research participants

Policy information about studies involving human research participants and Sex and Gender in Research.

Reporting on sex and gender	Study sample came from a subset samples from an earlier study - Carissimo G, X et al. Nat Commun. 2020;11(1):5243. Non-severe and severe samples were randomly selected to avoid bias. In total, we tested 15 non-severe (13 males) and 10 severe (8 males).
Population characteristics	Subset samples from a clinical study in Singapore - Severe COVID-19 were older and higher proportion were hypertensive based on medical records.
Recruitment	This was an observational cohort study of patients with PCR-confirmed COVID-19 who were admitted to the National Centre for Infectious Diseases, Singapore. All patients with COVID-19 in Singapore, regardless of the severity of infection, are admitted to isolation facilities until clinical recovery and viral clearance. For the current study, subset of samples were randomly selected from the original cohort
Ethics oversight	Ethics approval was obtained from the Domain Specific Review Board, National Healthcare Group (study ID: 2012/00917 and E/2016/00982). All participants provided written informed consent.

Note that full information on the approval of the study protocol must also be provided in the manuscript.

Field-specific reporting

Please select the one below that is the best fit for your research. If you are not sure, read the appropriate sections before making your selection.

☒ Life sciences ☐ Behavioural & social sciences ☐ Ecological, evolutionary & environmental sciences

For a reference copy of the document with all sections, see nature.com/documents/nr-reporting-summary-flat.pdf

Life sciences study design

All studies must disclose on these points even when the disclosure is negative.

Sample size	No sample size calculation was done. Sample numbers were based on sample availability and decision to investigate with current sample numbers were based on previous clinical research that provided evidence demonstrating significant differences between severe and non-severe cases. Carissimo G, X et al. Nat Commun. 2020;11(1):5243
Data exclusions	No data was excluded from analyses, all available samples were tested
Replication	All samples were tested in duplicate. Cell culture work was performed twice.
Randomization	Samples were from previously published manuscript (PMID: 33067472). For in vitro human endothelial cells work, we randomly picked severe and non-severe cases
Blinding	Investigators were not blinded. This is an observational study comparing severe and non-severe COVID-19

Reporting for specific materials, systems and methods

We require information from authors about some types of materials, experimental systems and methods used in many studies. Here, indicate whether each material, system or method listed is relevant to your study. If you are not sure if a list item applies to your research, read the appropriate section before selecting a response.

Materials & experimental systems

n/a	Involved in the study	
☐	☒	Antibodies
☒	☐	Eukaryotic cell lines
☒	☐	Palaeontology and archaeology
☒	☐	Animals and other organisms
☐	☒	Clinical data
☒	☐	Dual use research of concern

Methods

n/a	Involved in the study	
☒	☐	ChIP-seq
☒	☐	Flow cytometry
☒	☐	MRI-based neuroimaging

Antibodies

Antibodies used	Anti MPO (DY3174), Anti Syndecan -1(DY2780), Anti Glypican-1 (DY4519-05). From R&D system
Validation	Certificate of Analysis - MPO (LotP196836); syndecan -1(P234272); Glypican-1 (P289686)

Clinical data

Policy information about clinical studies

All manuscripts should comply with the ICMJE guidelines for publication of clinical research and a completed CONSORT checklist must be included with all submissions.

Clinical trial registration	For COVID-19 blood/plasma collection, “A Multi-centred Prospective Study to Detect Novel Pathogens and Characterize Emerging Infections (The PROTECT study group)”, a domain specific review board (DSRB) evaluated the study design and protocol, which was approved under study number 2012/00917 the National Healthcare Group (NHG).
Study protocol	This is not a clinical trial
Data collection	Healthcare settings Singapore
Outcomes	Observational study
