# Supplementary material for: Myeloperoxidase inhibition may protect against endothelial glycocalyx shedding induced by COVID-19 plasma
Source: Commun Med (Lond). 2023 May 5;3:62. doi: 10.1038/s43856-023-00293-x (PMC10160718; doi:10.1038/s43856-023-00293-x)
Supplement: Supplementary file 2 — Description of Additional Supplementary Files [file 43856_2023_293_MOESM2_ESM.pdf]

Description of Additional Supplementary Files

File Name: Supplementary Data

Description: Figure 1 - 5 dataset
