# Supplementary material for: Myeloperoxidase inhibition may protect against endothelial glycocalyx shedding induced by COVID-19 plasma
Source: Commun Med (Lond). 2023 May 5;3:62. doi: 10.1038/s43856-023-00293-x (PMC10160718; doi:10.1038/s43856-023-00293-x)
Supplement: Supplementary file 4 — Supplementary Information [file 43856_2023_293_MOESM4_ESM.pdf]

Myeloperoxidase inhibition may protect against endothelial glycocalyx shedding induced by COVID-19 plasma

Andrew Teo [1,2,3*], Louisa L Y Chan[1], Christine Cheung[1,4], Po Ying Chia[1,3,5], Sean Wei Xiang Ong[3,5], Siew Wai Fong [6], Lisa F P Ng[6,7,8], Laurent Renia[1,6,9], David Chien Lye[1,3,5,10], Barnaby Edward Young[1,3,5] Tsin Wen Yeo [1,3,5]

[1] Lee Kong Chian School of Medicine, Nanyang Technological University, Singapore, Singapore

[2] Department of Medicine, The Doherty Institute, University of Melbourne, Melbourne, Australia

[3] National Centre for Infectious Diseases, Singapore, Singapore

[4] Institute of Molecular and Cell Biology, Agency for Science and Technology and Research (A*STAR), Singapore, Singapore

[5] Department of Infectious Diseases, Tan Tock Seng Hospital, Singapore, Singapore

[6] A*STAR Infectious Diseases Lab, Agency for Science and Technology and Research (A*STAR), Singapore, Singapore

[7] Institute of Infection, Veterinary and Ecological Sciences, University of Liverpool, Liverpool, UK

[8] National Institute of Health Research, Health Protection Research Unit in Emerging and Zoonotic Infections, University of Liverpool, Liverpool, UK

[9] School of Biological Sciences, Nanyang Technological University, Singapore, Singapore

[10] Yong Loo Ling School of Medicine, National University of Singapore, Singapore, Singapore

* Corresponding author: andrewcc.teo@ntu.edu.sg

Supplementary Table 1. Correlations between myeloperoxidase levels and myeloperoxidase activities with age, days posts symptoms onset and white blood cells count at enrolment

Acute phase myeloperoxidase levels vs host's variables	Spearman, R	P	Acute phase myeloperoxidase activities vs host's variables	Spearman, R	P
Age, n =25	0.09	0.65		-0.11	0.60
Days post symptoms onset, n =25	0.34	0.09		-0.30	0.14
Lymphocytes, n=24	-0.34	0.09		-0.07	0.72
Monocytes, n=24	0.12	0.56		-0.21	0.34
Neutrophils, n=24	**0.54**	**0.006**		0.01	0.99
Platelets, n=24	0.02	0.90		-0.26	0.21
White blood cells, n=24	0.39	0.054		-0.05	0.83
Neutrophils : lymphocytes, n=24	**0.50**	**0.011**		0.02	0.93

Rho determined by Spearman correlation coefficient, P <0.05 considered significant, in bold.

Supplementary Table 2. Correlation between mediators associated with syndencan-1 and glypican-1 shedding.

Variables	Acute phase mediators vs acute phase syndecan-1		Convalescent phase mediators vs convalescent phase syndecan-1		Acute phase mediators vs acute phase glypican-1		Convalescent phase mediators vs acute phase glypican-1	
	R	P value	R	P value	R	P value	R	P value
CRP, n = 22	**0.76**	**<0.0001**			0.32	0.15		
MPO, n = 25	**0.42**	**0.03**	**0.39**	**0.052**	0.30	0.15	0.08	0.69
MPO activities, n = 25	**0.48**	**0.003**	**0.47**	**0.02**	0.17	0.41	0.05	0.80
IFN-γ, n=25	-0.25	0.23	-0.05	0.83	0.22	0.29	0.13	0.53
IL-10, n=25	**-0.42**	**0.037**	-0.377	0.069	0.01	0.96	-0.01	0.96
IL-2, n =25	0.05	0.81	-0.36	0.078	-0.01	0.97	**-0.50**	**0.012**
IL-6, n=25	0.26	0.20	**0.44**	**0.028**	-0.04	0.82	0.18	0.40
IP-10, n=25	**0.67**	**0.0003**	0.20	0.36	**0.41**	**0.044**	-0.17	0.42
MIP-1 α, n=25	0.05	0.81	0.13	0.54	0.32	0.11	0.22	0.30
MIP-1 β, n=25	0.11	0.60	0.11	0.60	0.08	0.68	0.08	0.70
TNF- α, n =25	0.01	0.95	-0.20	0.35	0.08	0.67	-0.38	0.063
VEGF-α, n=25	0.08	0.68	0.09	0.67	0.12	0.56	0.11	0.59
VEGF-β, n=25	0.27	0.18	0.14	0.53	0.15	0.47	0.27	0.20

Rho determined by Spearman correlation coefficient, P <0.05 considered significant, in bold.

Abbreviations: CRP; C-reactive protein, MPO; myeloperoxidase, IFN; interferon, IL; interleukin, MIP; macrophage inflammatory protein, TNF; tumour necrosis factor, VEGF; vascular endothelial growth factor
